# An improved respiratory syncytial virus neutralization assay based on the detection of green fluorescent protein expression and automated plaque counting

**DOI:** 10.1186/1743-422X-9-253

**Published:** 2012-10-31

**Authors:** Yvonne van Remmerden, Fang Xu, Mandy van Eldik

**Affiliations:** 1 Department of Vaccine Research, Vaccinology, National Institute for Public Health and the Environment, Bilthoven, The Netherlands; 2 Nobilon International BV, Boxmeer, The Netherlands; 3 Present address: Merck/MSD Animal Health, Boxmeer, The Netherlands; 4 Present address: Crucell, Leiden, The Netherlands

**Keywords:** RSV, Virus neutralization, EGFP

## Abstract

**Background:**

Virus neutralizing antibodies against respiratory syncytial virus (RSV) are considered important correlates of protection for vaccine evaluation. The established plaque reduction assay is time consuming, labor intensive and highly variable.

**Methods:**

Here, a neutralization assay based on a modified RSV strain expressing the green fluorescent protein in combination with automated detection and quantification of plaques is described.

**Results:**

The fluorescence plaque reduction assay in microplate format requires only two days to complete and is simple and reproducible. A good correlation between visual and automated counting methods to determine RSV neutralizing serum antibody titers was observed.

**Conclusions:**

The developed virus neutralization assay is suitable for high-throughput testing and can be used for both animal studies and (large scale) vaccine clinical trials.

## Background

Human Respiratory Syncytial virus (RSV) is a non-segmented, negative-strand RNA Virus and is a member of the *Paramyxoviridae*. RSV is the most common cause of lower respiratory tract infections in infants and elderly [1-3]. The majority of infected subjects develop upper respiratory tract infection, but in severe cases RSV infection can result in bronchiolitis, pneumonia and mortality. RSV is increasingly being recognized as a significant cause of morbidity and mortality in the elderly, with an impact approaching that of non-pandemic influenza [4]. The high disease burden indicates an urgent need for an efficacious vaccine against RSV, however despite numerous approaches aimed at its development there is currently no licensed vaccine available. Major obstacles include the legacy of RSV vaccine enhanced disease and early age of RSV infection. Promising vaccine candidates currently in preclinical or clinical testing include live- attenuated recombinant RSV, chimeric viruses and replication-defective vectors [5].

Accordingly, there is need for reliable and high-throughput tests to determine the immunogenicity of vaccine candidates in both animal models and large-scale clinical trials. While there are many methods providing information on different aspects of the immune response, the neutralization assay for RSV antibodies remains the most reliable correlate of protection [6,7]. The fact that currently the only available prophylactic agent against RSV infection is a virus neutralizing monoclonal antibody preparation underscores the importance of virus neutralizing antibodies in protection from severe disease [8].

The power of the neutralization assay lies in its ability to detect biologically active antibodies. In the conventional neutralization assay based on plaque reduction, stained plaques are counted manually, often with the aid of a microscope. This is a laborious and time consuming procedure, while operator bias makes it less suitable for clinical trials that involve testing large numbers of samples in multiple centres. Therefore, improvement of the assay is required to optimize inter-lab testing and throughput. Reports on optimization of the virus neutralization (VN) assay include visualization of plaques by biochemical staining of monolayers [9], but still this requires manual counting of plaques. Another study reports the use of an automated plaque counting method that still requires immunostaining [10]. None of these approaches circumvent all constraints of the traditional assay format.

Recent reports have shown that virus constructs containing the (E)GFP gene can be used for more easy and quick determination of virus neutralizing activity [11-13]. This finding enables the development of an assay system that can be automated, rendering it less labor intensive and operator dependent, thereby making the assay more suitable for standardization and high throughput purposes. Importantly, this would also facilitate validation of the assay according to ICH guidelines, thus enabling its use in multicenter clinical trials. In this report, the development of such an assay for detection of RSV neutralizing antibodies is described. The assay design is based on a recombinant RSV construct expressing EGFP from the viral genome in combination with automated fluorescent plaque counting. Our results show good correlation between visual and automated counting methods to determine RSV neutralizing serum antibody titers.

## Results

### Replication of recombinant RSV harbouring the EGPF gene

**Figure 1:**
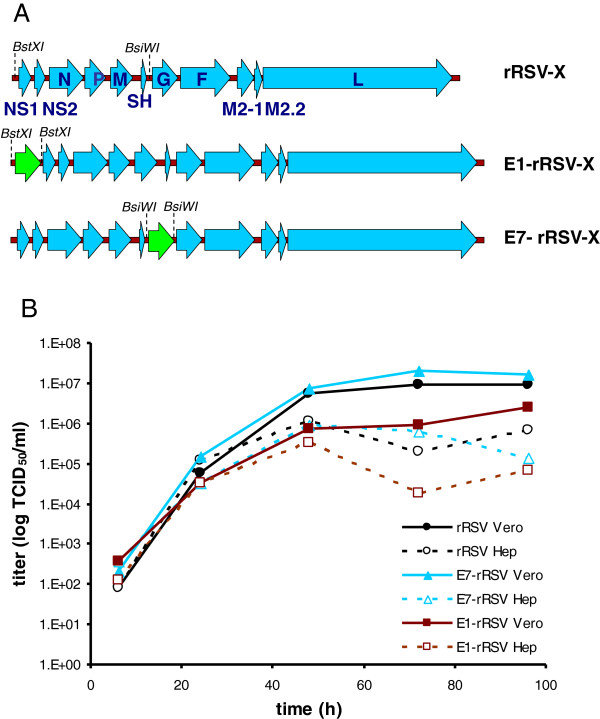
**A)Schematic diagram of constructed recombinant RSV (rRSV-X)expressing EGFP gene in the first(E1-rRSV-X)and the seventh(E7-rRSV-X)position in the virus genome and the positions of the restriction enzymes in the RSV-X genome used for insertion of EGFP.** Blue: RSV gene; green: EGFP gene. **B**) Replication kinetics of recombinant RSV viruses E1-rRSV-X, E7-rRSV-X and the parental rRSV-X in Vero and Hep-2 cells. Cells were infected at a multiplicity of infection (MOI) of 0.1 with the rRSVs. At the indicated time points, cells and supernatants were collected and virus titers were determined. Closed lines represent growth on Vero cells; dashed lines represent growth on Hep-2 cells.

Using reverse genetics, the EGFP gene was inserted either at the 3’ proximal site (E1-rRSV-X) or between the SH and G gene (E7-rRSV-X) of RSV-X (Figure 1A). EGFP was shown to be expressed in virus infected cells, readily detectable using a fluorescence microscope (data not shown). The growth kinetics of the recombinant viruses was compared with their parental recombinant wild-type virus (rRSV-X). For this purpose, Vero and Hep-2 cells were infected with a multiplicity of infection (MOI) of 0.1 (Figure 1B). Growth of E7-rRSV-X on either Vero or Hep-2 cells was indistinguishable from the parental rRSV-X strain, reaching peak titers of 10^7^ TCID_50_.ml^-1^ at 72 hrs post infection of Vero cells. Construct E1-rRSV-X was attenuated compared to rRSV-X and E7-rRSV-X. Therefore, E7-rRSV-X was selected for use in development of the fluorescent plaque assay.

### Virus neutralization (VN) assay using E7-rRSV

**Figure 2:**
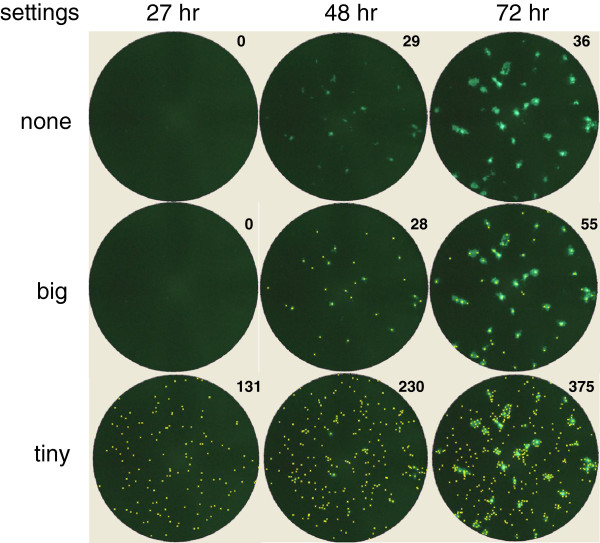
**Fluorescent images of Vero cells infected with E7-rRSV-X over time.** Initial input is 115 PFU per well. The images show plaques produced by RSV expressing EGFP. At the indicated time points, plaques were detected after 27 hrs, 48 hrs and 72 hrs. in a fluorescence Elispot reader, manual counted (upper panel) and counted using the AID EliSpot Software 'algorithm C' with emphasis settings were set on ”big” (middle panel) or “tiny” (lower panel). Each yellow ‘x’ represents one plaque counted by the imager software.

**Figure 3:**
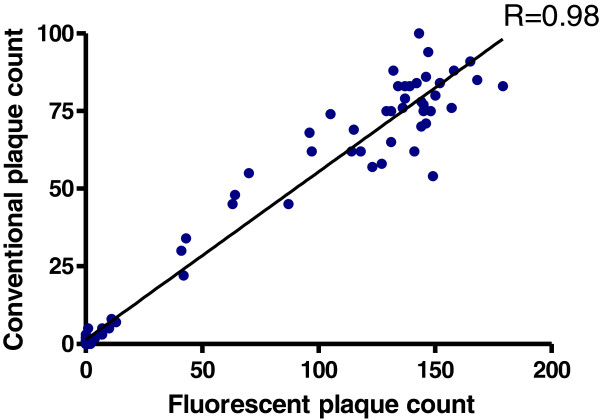
**Correlation of automated and conventional plaque counting.** Scatter plots of counted plaques by the automatic counting of fluorescent plaques (X-axis) and manual counting of the same plaque immunostained (Y-axis). Plaques were counted 48 hrs after infection using the ‘big’ settings.

**Figure 4:**
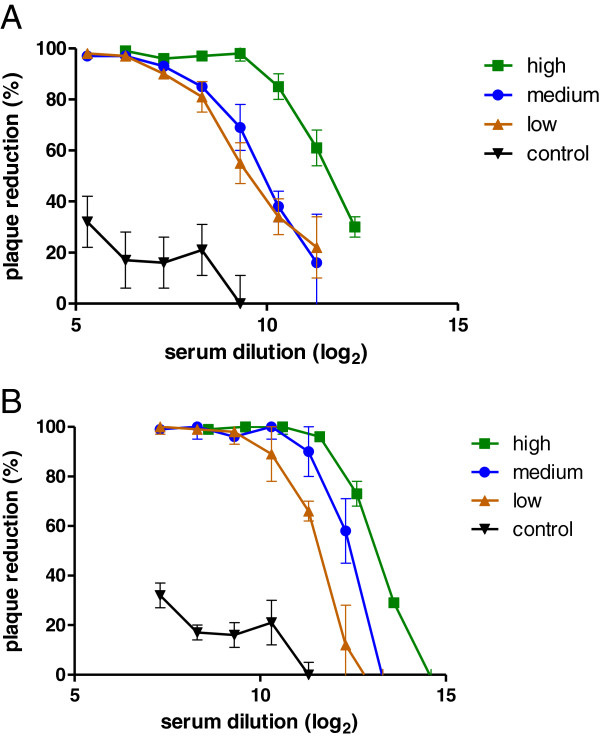
**Sample graph of neutralization assay results with human reference sera.** Virus neutralizing graphs by polyclonal anti-human reference sera (high, medium and low control) [15] obtained through the NIH Biodefense and Emerging Infections Research Resources Repository, NIAID, NIH in the absence (**A**) or presence (**B**) of 10% guinea pig serum. As negative control, control serum from naive cotton rats was included because of the lack of availability of negative human reference serum. Plaque reduction titers were calculated by regression analysis of the inverse dilution of serum that provided a 60% plaque reduction titer compared to control wells in the absence of serum. Results are means ± SD (triplicate). Settings of fluorescence reader are as described for Figure 3.

First, an optimal virus inoculum that resulted in clear and distinct fluorescent virus plaque formation after one to three days of incubation was determined. The established optimum of 30 to 300 PFU (data not shown) is in the same order of magnitude as used in the conventional RSV assay as well as in other conventional virus neutralization assays. An inoculum of 115 PFU was used as standard for all further assays. Analysis of EGFP expression over time showed that plaques of E7-rRSV-X infected cells are already visible after 27 hours (Figure 2). However, their size was small and the relatively low fluorescence intensity of infected cells resulted in contrast levels that are too low to allow for accurate automated plaque counting. At 72 hrs post infection large differences in plaque size were observed, complicating the establishment of fixed settings on the equipment required for automated plaque counting. An incubation time of 48 hrs proved to be optimal for data acquisition and quantification of plaques; at this time point small, individual and bright fluorescent plaques with little variation in size were observed. Next, the correlation was assessed between automated counting of fluorescent plaques and manual counting of the same plaques immunostained (Figure 3). A high correlation was observed when comparing plaques against the rE7-rRSV virus by both automated fluorescent and manual immunostained plaque counting (R=0.98). Serum neutralizing antibodies titers tend to be higher when assayed in the presence of guinea pig complement [14]. Therefore, the effect of complement on the fluorescent virus neutralization assay was assessed using RSV specific human reference sera obtained through the NIH Biodefense and Emerging Infections Research Resources. Figure 4 shows the effect of guinea pig complement on the plaque reduction capacity of the serum. The obtained serum neutralizing antibody titers for the low, medium and high human reference serum pools were 11.4, 12.3 and 13.0 log_2_ respectively in the presence of complement and 9.1, 9.6 and 11.3 log_2_ in the absence of complement. These data correspond well to the titers described by Yang *et al*. [15]. Although the measured neutralizing antibody titers were indeed higher in the presence of complement, its addition also decreased the discriminative power of the assay as it reduced differences in virus neutralizing titers between control and reference sera. In addition, complement seemed to inhibit RSV replication in this infection system, as it required a two-fold higher input of virus to yield similar plaque counts. Moreover, the presence of complement increased inter-assay variation (data not shown). No effect on virus neutralizing titers was observed when the initial RSV input was either increased or decreased a two-fold (data not shown).

**Figure 5:**
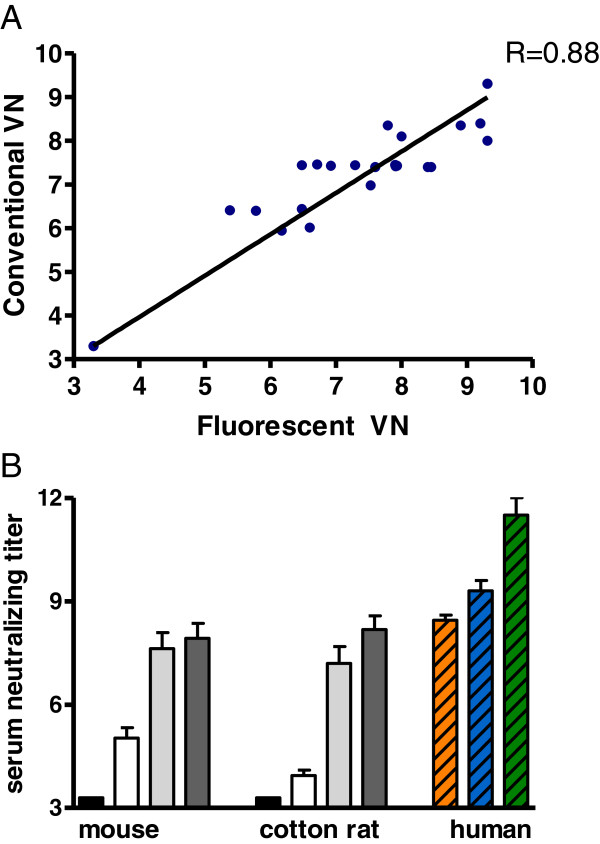
**Fluorescent virus neutralizing antibody plaque titers.A**) Scatter plots of virus neutralization (VN) titers obtained by automatically counted plaques of fluorescence E7-rRSV versus manually coloured and counted plaque reduction of the parental rRSV. **B**) Serum antibody neutralization titers of cotton rat, mouse and human serum samples obtained with the fluorescent virus neutralizing antibody assay. Serum neutralizing titers from different experiments are depicted. Animals were immunized with RSV vaccine candidates [16]. Pre-immune serum was obtained at day 0 (black bars) and were at the lower limit of detection. Mice were immunized at day 0 and day 28 with 10^6 RSV- ΔG and serum was obtained at day 28 (light gray) and at day 51 (gray) (n=6 per group). Cotton rats were immunized at day 0, day 14 and day 28 with 10^5 RSV-ΔG and serum was obtained at day 28 (light gray) and at day 51 (gray) (n=5 per group). As reference, serum from animals immunized with formalin-inactivated RSV (white) are included (n=5 per group). The human reference human sera: high (green), medium (blue) and low (orange) were included as standards [15]. Error bars indicate standard deviation.

Next, virus neutralization using conventional, manual counting of virus plaques using the parental rRSV construct was compared with automated fluorescent plaque reduction quantification of rE7-rRSV, also to determine the influence of EGFP insertion into the viral genome (Figure 5A). There was a good agreement between the titers obtained by both assays (R= 0.88). Finally, we wanted to determine if the automated fluorescent virus plaque reduction assay is appropriate for use in immunogenicity studies in both cotton rat and mouse models of RSV infection. To this aim, virus neutralizing antibody titers of cotton rat and mouse sera were determined by the newly developed plaque reduction assay, in the absence of complement (Figure 5B). Pre-immune sera from both cotton rats and mice were negative while infection with a recombinant RSV vaccine candidate (rRSV∆G) [16] resulted in high virus neutralizing antibody titers with low variation between animals within a group. For comparison, the serum neutralizing antibody titers obtained for the human reference serum pools are included. These results suggest that the fluorescent plaque reduction setup is such that the assay can be performed independent of the host of the serum.

## Discussion

We describe the development of a simple and rapid micro neutralization assay for RSV based on a recombinant RSV construct expressing the EGFP protein. To this aim, we generated two recombinant RSV viruses: one harbouring EFGP at the 3’ proximal position (E1-rRSV-X) and one harbouring EGFP between the SH and G gene (E7-rRSV-X). Both recombinant RSV clones were constructed with an EGPF gene flanked by RSV gene start and stop signals, ensuring the expression of EGFP in virus infected cells. E7-rRSV-X was selected to further develop the neutralization assay since growth properties of this virus on Vero cells were indistinguishable from the parental rRSV-X virus, while E1-rRSV-X was shown to be attenuated. This suggests that positioning the EGFP gene at the 3’ end of the viral genome, i.e. before the first gene, most likely negatively affects transcription and, by extension, virus growth, a phenomenon that has been described previously [17-19]. Using the current assay, virus neutralization titers can be determined within 2 days of incubation without requiring fixation, staining and manual counting of plaques. The results showed a strong correlation between automated EFGP and manual counting of immunostained plaques for the detection of RSV neutralizing antibody titers.

Serum neutralizing antibody titers to respiratory syncytial virus (RSV) are higher when assayed in the presence of guinea pig complement [14,15]. In the current assay, the addition of complement also resulted in increased virus neutralizing titers compared to incubation without complement. However, the observation that the amount of virus needed to obtain similar plaque counts in the presence of complement suggest a more complex, possibly confounding influence of complement on the outcome of the virus neutralization assay. While apparently improving the sensitivity of the assay, the opposite may be happening simultaneously through a direct inhibitory effect of complement, as suggested by the need for higher MOI levels in the test. By directly eliminating a portion of the input virus, thereby decreasing the number of targets for VN antibodies, complement may actually decrease the sensitivity of the assay. Therefore we chose to omit the use of complement in our neutralization assay. These findings are in agreement with Yoder *et al*. [14], who reported that addition of complement did not enhance the ability to detect increased antibody titers after natural infection in humans and have not found evidence to support its use in the assay. As these authors, we believe that the addition of complement introduces an additional, possibly confounding and variable in the performance of the test and we would advocate that RSV neutralization assays be carried out without complement, in order to provide a more standardized and robust assay.

The main advantage of the currently developed method is that the most time consuming, labour intensive parts of the RSV neutralization assay (i.e. fixation, staining and plaque counting) are replaced by computerized image scanning and analysis. These features result in a faster and more efficient assay and ensure less subjective results through the exclusion of operator bias. Another advantage of the automated counting system is that it is capable of detecting and differentiating plaques of different morphology. This feature could be used to detect serum neutralizing antibodies against other plaque forming viruses, although this requires recombinant viruses harbouring EGFP. In addition, the fluorescence ELISPOT reader can also be used to detect virus infected cells by staining with fluorescently labelled antibodies as an alternative for the classical plaque forming unit (PFU) assay. In fact, we have developed such a fluorescent plaque forming assay for quantification of RSV that correlates well with the classical PFU assay (data not shown).

Using the fluorescence ELISPOT reader, a plate can be processed within 5 min, including reading, counting and analysis. Robotic automation of plate loading could be introduced for high throughput purposes. These assets would render the fluorescent neutralization assay a valuable tool for analysis of humoral immune responses in vaccine studies and for serological studies in naturally infected hosts. Taken together, our virus neutralization assay is a major improvement compared to other described micro neutralization assays [9,10] which still require either manual plaque counting or immunostaining. Recently, a flow cytometry-based assay to assess RSV-specific neutralizing has been described [20]. As our method, this method is based on the expression of EGPF by the viral genome and can be performed within two days. However this assay requires manual treatments prior to analysis while our fluorescence-based plaque reduction assay can be measured directly.

## Conclusions

We have used a recombinant RSV virus expressing EGPF to develop a virus neutralization assay; this eliminates the time consuming fixation and staining steps as well as manual plaque counting. This fluorescence plaque reduction assay in microplate format requires only two days to complete and is simple, reproducible and suitable for high-throughput analyses. The developed virus neutralization assay can be used for the analysis of sera from both animal studies and (large scale) vaccine clinical trials. Furthermore, our data indicate that the fluorescent plaque reduction assay can be utilized to measure neutralizing antibody titers against a broad range of plaque-forming viruses.

## Methods

### Viruses, cells and serum

Vero cells (CCL-8, American Type Culture Collection (ATCC)) were cultured (37°C, 5% CO_2_) in M199 medium (Invitrogen) supplemented with heat-inactivated 5% foetal bovine serum (FBS, Hyclone) and PSG (100 units of penicillin, 10 μg of streptomycin and 292 μg of L-glutamine/ml, Invitrogen). Hep-2 (CCL-23, ATCC) were cultured in DMEM medium (Invitrogen) with 10% FBS and PSG. The clinical isolate RSV-X [Genbank FJ948820], a RSV sero group A strain, was used as the basis to create recombinant RSV by reverse genetics [16]. RSV specific human reference sera was obtained through the NIH Biodefense and Emerging Infections Research Resources Repository, NIAID, NIH: polyclonal anti-human Respiratory Syncytial Virus (antiserum, human), medium control, NR-4022; polyclonal anti-human Respiratory Syncytial Virus (antiserum, human), high control, NR-4021 and polyclonal anti-human Respiratory Syncytial Virus (antiserum, human), NR-4020 designated as low control. Mouse and cotton rat serum samples were derived from immunization experiments with RSV vaccine candidates performed at the RIVM.

### Construction of RSV plasmids expressing the EFGP gene

The plasmid containing the complete antigenomic sequence (rRSV-X) of the clinical isolate RSV-X [Genbank FJ948820] [16] was used as basis to construct EGFP-rRSV-X. First, a full-length rRSV cDNA clone with EGPF at position 7 of the viral genome was constructed. The EGFP gene was amplified by PCR from the pEGFP plasmid (Clonetech) during which gene start and stop signals of RSV G, both preceded by *BsiW* I were introduced. Subsequently, the EGFP amplicon was digested with *BsiW* I, and cloned into the *BsiW* I site of the RSV cDNA vector (n4667-4672). The resulting plasmid was designated E7-rRSV-X.

Second, a full-length rRSV cDNA vector with EGPF at a natural occurring *BstX* I site (n35-46) in the leader region before the first gene of the viral genome was constructed. To this end, the EGFP gene was amplified from pEGFP flanked by the gene start signal of RSV G and the gene stop signal of N, each preceded by *BstX* I. This amplification product was cloned into a subclone harbouring the RSV leader sequence along with the NS1, NS2 and N gene using *BstX* I. Subsequently, the RSV sequence with the EGFP gene of the newly constructed subclone then was swapped into the full length rRSV cDNA plasmid using the *Kpn* I and *Xma* I restriction sites. This plasmid was designated E1-rRSV-X.

### Recovery of recombinant viruses

Recovery of recombinant RSV harbouring the EGPF gene was performed as described before [16]. MVA-T7 infected Hep-2 cells were transfected using lipofectamine 2000 with 1.6 μg of the recombinant full length plasmids and 1.6 μg pcDNA6-A2-N, 1.2 μg pcDNA3-A2-P, 0.4 μg pcDNA6-A2-L, and 0.8 μg pcDNA6-A2-M2. After 3 days at 32°C, cells were scraped and used to infect fresh cultures of Vero cells grown in DMEM + 1% FCS + PSG. Recovered virus was propagated 4 to 5 times in Vero cells to obtain high virus titer stocks.

### Fluorescence-based plaque reduction assay

Two-fold serial dilutions starting at 1:10 of serum were prepared in virus diluent (DMEM supplemented with 1% FCS and PSG). Serum was first incubated for 30 min at 56°C, and then serum dilutions were mixed with an equal volume of virus (115 plaques/well) and incubated for 1 hr at 37°C. If sera were tested in the presence of 10% guinea pig complement (Cederlane Laboratories), this was added to the serum prior to the addition of virus. Vero cell monolayers, prepared in 96-well plates, were infected by spin inoculation with 50 μl/well (in triplicate) of the serum/virus mixture. After centrifugation for 1 h at 700x*g* and additional 1 hr incubation at 37°C, supernatant was removed and cells were overlaid with 1.0% methyl cellulose in DMEM supplemented with 1% FCS and PSG. Hereafter, the microtiter plates were incubated at 37°C and 5% CO_2_. At the indicated time points, plaques were detected in a fluorescence Elispot reader (AID iSpot FluoroSpot Reader System - Autoimmun Diagnostika GmbH Germany) and counted using the AID EliSpot Software 'algorithm C' with emphasis settings were set on ”tiny or were set on “big”. Plaque reduction titers were calculated by regression analysis of the inverse dilution of serum that provided a 60% plaque reduction titer compared to control wells incubated without serum.

### Immunostaining of plaques

Vero cells were infected with the serum/virus mixture as described above. After incubation for two days at 37°C, the cells were immunostained. To this aim, the overlay was first removed and the cells were fixed with 80% acetone for 30 minutes at room temperature. After incubation with monoclonal L9 anti-RSV G [21] followed by goat anti-mouse-IgG-PO (Invitrogen), plaques were visualized using the True Blue TMB peroxidase substrate (KPL).

## Competing interests

The authors declare they have no competing interests.

## Authors' contributions

Design and conception of the study and drafted the manuscript (MNW), development of the methods and co-drafted the manuscript (YVR), assisted in development of the assay (ME), constructed the recombinant clones (XF), manuscript preparation and review (WH, JH). All authors approved the final version of the manuscript.
